# Periodic Limb Movements during Sleep in Acute Stroke: Prevalence, Severity and Impact on Post-Stroke Recovery

**DOI:** 10.3390/jcm12185881

**Published:** 2023-09-10

**Authors:** Panagiotis Plomaritis, Aikaterini Theodorou, Vasiliki Michalaki, Maria-Ioanna Stefanou, Lina Palaiodimou, Georgia Papagiannopoulou, Vasiliki Kotsali-Peteinelli, Marianna Bregianni, Elissavet Andreadou, Georgios P. Paraskevas, Sotirios Giannopoulos, Georgios Tsivgoulis, Anastasios Bonakis

**Affiliations:** 1 Second Department of Neurology, “Attikon” University Hospital, School of Medicine, National and Kapodistrian University of Athens, 12462 Chaidari, Greece; pjdoc223@gmail.com (P.P.); katetheo24@gmail.com (A.T.); vanessamichalaki@yahoo.com (V.M.); marianna421@hotmail.co.uk (M.-I.S.); lina_palaiodimou@yahoo.gr (L.P.); georgiapap22@hotmail.com (G.P.); vkotsali@gmail.com (V.K.-P.); marianna.breg@gmail.com (M.B.); geoprskvs44@gmail.com (G.P.P.); sgiannop@uoi.gr (S.G.); tsivgoulisgiorg@yahoo.gr (G.T.); 2 First Department of Neurology, “Eginition” University Hospital, School of Medicine, National and Kapodistrian University of Athens, 11528 Athens, Greece; elisandread@gmail.com; 3 Department of Neurology, University of Tennessee Health Science Center, Memphis, TN 38163, USA

**Keywords:** acute stroke, periodic limb movements during sleep, polysomnography, predictors, stroke severity, stroke type, functional outcome

## Abstract

Background: Periodic Limb Movements during Sleep (PLMS) have been described to be frequently present in stroke patients. We aimed to evaluate the prevalence and severity of PLMS in acute stroke patients and clarify the association between PLMS and coexisting Sleep Disordered Breathing (SDB). Additionally, we focused on identifying variables that could independently predict the presence of PLMS in patients with acute stroke. The potential impact of PLMS on stroke outcome at three months was investigated as well. Methods: In this study, we performed overnight polysomnography on consecutive stroke patients within 72 h from symptom onset. Data regarding clinical and imaging characteristics were prospectively collected. National Institute of Health Stroke Scale (NIHSS), modified Rankin Scale (mRS) and Epworth-Sleepiness Scale (ESS) were used to evaluate stroke severity on admission, stroke outcome at three months and history of daytime sleepiness, respectively. We documented PLMS and SDB using standard polysomnography criteria. Results: We prospectively assessed 126 patients with acute stroke [109 with ischemic and 17 with hemorrhagic stroke, mean age 60 ± 11 years, 68% men, median NIHSS score on admission: 3 (IQR: 2–7)]. The overall rate of PLMS in our cohort was 76%, and the rate of SDB among patients with PLMS was 83%. PLMS detection rates differed significantly (*p*-value: <0.001) according to SDB, with PLMS prevalence increasing with greater SDB severity. SDB could independently (OR:4.869, 95% CI: 1.884–12.784, *p*-value: 0.001) predict the presence of PLMS in the acute stroke phase in multivariable analyses adjusting for potential confounders. Moreover, baseline stroke severity (NIHSS-score increase in per-1 point: OR: 0.819, 95% CI: 0.737–0.895, *p*-value < 0.001) and PLMS (OR:0.099, 95% CI: 0.009–0.482, *p*-value = 0.015) were significantly associated with the likelihood of excellent functional outcome (mRS-scores: 0–1) at 3 months. Conclusion: The common presence of mostly severe PLMS in patients with acute stroke and their negative effect on stroke outcomes point out the necessity for early PLMS detection and treatment.

## 1. Introduction

Periodic limb movements during sleep (PLMS) are limb movements (LMs) characterised by the stereotypic dorsiflexion/extension of the big toe, dorsiflexion at the ankle, and sometimes flexion at the knee and hip joints [1]. They occur repetitively and spontaneously during sleep, leading to sleep fragmentation, sympathetic overactivity (increase in heart rate and blood pressure) and daytime sleepiness [2].

PLMS are detected through polysomnography by means of surface electromyography of the anterior tibialis muscle. The number of periodic limb movements (PLMs) occurring per hour of total sleep time constitutes the PLMS index (PLMSI) [1]. According to the American Academy of Sleep Medicine (AASM) criteria, PLMS are considered clinically significant when PLMSI is at least 15 per hour [1]. PLMs are mostly observed as part of restless leg syndrome (RLS) but can also occur in other sleep disorders such as REM (rapid eye movement) behaviour disorder (RBD), narcolepsy and sleep-disordered breathing (SDB) [2]. Furthermore, patients with Parkinson’s disease (PD), multiple system atrophy (MSA) or other medical conditions such as end-stage renal disease and depression may also exhibit PLMs [2]. On the other hand, periodic limb movement disorder (PLMD) constitutes a primary sleep disorder which is characterised by the presence of clinical sleep disturbance or impaired daytime function that cannot be attributed to another underlying cause [2].

There is already evidence connecting PLMS with the risk of cardiovascular and cerebrovascular disease [3,4]. The possible implicated mechanisms include both sympathetic overactivity and inflammatory pathways [5,6,7]. The severity of PLMS among patients with cerebrovascular accidents (CVA) is worse than in the general population, and the prevalence of CVA is higher among patients with PLMS [4]. In addition, there are limited data connecting severe PLMS with stroke recurrence [8]. Furthermore, current studies demonstrate a high prevalence of PLMS in stroke survivors, which appears to range from 32% to 48% [9]. On the other hand, PLMs can also occur due to respiratory events [apnea, hypopnea or RERA (respiratory effort-related arousal)] in stroke patients with SDB. SDB is the only sleep disorder so far described to have a high prevalence among acute stroke patients and a negative impact on functional recovery [10,11,12]. Therefore, distinguishing between SDB-associated (also known as Respiratory-Related Leg Movements, RRLS) and “genuine” PLMS seems to be necessary in order to evaluate the potential effect of PLMS on stroke outcomes and implement the appropriate treatment after stroke.

The goal of our prospective study was to assess the prevalence and severity of PLMS in the hyperacute stroke phase, taking into account the possible coexisting SDB of our patients, and to evaluate potential associations of PLMS with vascular risk factors, stroke characteristics and functional outcomes at three months.

## 2. Materials and Methods

Access to data supporting the results of our study can be reasonably requested from the corresponding author. We performed this study according to the STROBE guidelines for reporting observational research [13].

### 2.1. Study Design and Regulations

Before starting our prospective longitudinal study, we obtained institutional approval from the ethics committee of our hospital (Decision Number: EBD113/27-02-2018). Prior to enrollment, all patients or their legal representatives provided written informed consent.

### 2.2. Setting and Eligibility Criteria

We prospectively evaluated consecutive patients with acute stroke admitted to our stroke center (Second Department of Neurology of the National and Kapodistrian University of Athens, “Attikon” University Hospital, Athens, Greece) over a four-year period (May 2018–September 2022). All patients diagnosed with either acute ischemic stroke (AIS) or intracerebral hemorrhage (ICH) underwent overnight PSG during the first 72 h from symptom onset.

A detailed description of our study population was also reported in our previous study [14]. Exclusion criteria for this study included: (1) diagnosis of transient ischemic attack, (2) previous known history of SDB or PLMS with or without treatment with continuous positive airway pressure (CPAP), (3) presence of situations that would render cooperation with PSG unfeasible such as high stroke severity (NIHSS score >25 points), speech comprehension disorders or altered mental status, (4) presence of confounders that could interfere with measurements such as baseline oxygen saturation <95%, acute respiratory infection, stroke mimics including brain tumours, seizures and Todd’s paralysis and toxic–metabolic disturbances [15].

Further exclusion of patients from the study was based on the presence of invalid PSG data due to one of the following reasons: (1) insufficient total sleep time (3 h), (2) impossible evaluation of sleep stage, (3) >80% of total recording time had a poor airflow signal or a poor EMG signal from both anterior tibialis muscles, and (4) >70% of the data were lost in the recording [16].

Finally, patients who declined to participate in this study undergo PSG or failed to provide written informed consent were also excluded.

### 2.3. Data Collection

NIHSS score (National Institute of Health Stroke Scale) was used for the evaluation of stroke severity on admission (NIHSSadm) and at hospital discharge (NIHSSdis) [17]. A modified Rankin scale (mRS) score was used for the assessment of functional outcome at 3 months post index event [18]. History of vascular risk factors (arterial hypertension, diabetes mellitus, hyperlipidemia, atrial fibrillation, body mass index (BMI)/obesity, current smoking, history of myocardial infarction, congestive heart failure, history of excessive alcohol intake, history of stroke or TIA), demographics (sex, age) and acute reperfusion therapies in AIS were prospectively recorded for all patients.

We performed comprehensive diagnostic workup on all patients according to the American Heart Association (AHA) recommendations [19], which included (1) brain imaging with computed tomography (CT) or magnetic resonance imaging (MRI), (2) cervical and intracranial artery imaging with CT- or MR-angiography and ultrasonography (cervical duplex ultrasound and transcranial Doppler) performed by a certified neurosonologist, (3) cardiac imaging with transthoracic or transoesophageal echocardiography, (4) cardiac monitoring with a 12-lead electrocardiogram (ECG), and/or an ECG Holter (>24 h) performed by certified cardiologists, (5) routine blood and serum tests, as standard of care.

Stroke type (AIS or ICH) classification was made according to discharge diagnosis, and ischemic stroke aetiology was classified using the TOAST (Trial of ORG10172 in Acute Stroke Treatment) criteria [20]. Patients with embolic stroke of undetermined source (ESUS) were identified according to recent diagnostic criteria [21]. We prospectively followed all patients, evaluated their functional status at 90 days after symptom onset and captured functional outcomes 3 months after the index event as excellent (mRS scores of 0–1) or poor (mRS scores of 2–6). Assessment of all outcome events was carried out by attending-level stroke neurologists who were unaware of the PSG findings.

### 2.4. Sleep Evaluation

A questionnaire of the Epworth Sleepiness Scale (ESS) was used in every patient in order to evaluate the history of daytime sleepiness. An ESS score > 9 was considered abnormal [22,23]. A type 2 (unattended) overnight sleep study was performed on all patients in the hospital wards from 10 p.m. to 8 a.m. The device we used for this purpose was a Nox-A1 portable PSG system (Nox Medical, Inc., Reykjavik, Iceland), which included wireless pulse oximetry, body position sensor, respiratory inductive plethysmography, a nasal airflow/pressure sensor, an electrocardiogram, 3 submental electromyogram (EMG) channels, 2 electrooculogram (EOG) channels, six electroencephalogram (EEG) channels (F3-M2, F4-M1, C3-M2, C4-M1, O1-M2 and O2-M1) and 2 anterior tibialis EMG channels.

An apnea event was scored when an airflow decrement of ≥90% from the baseline was documented, lasting ≥10 s [24]. The continuous presence of respiratory effort during the event accounted for obstructive apnea, whereas the absence of respiratory effort throughout the event constituted a central apnea. When respiratory effort was absent during the first section of the event but appeared during the last section of it, apnea was characterised as mixed [25,26].

In this study, we documented a hypopnea event when airflow decreased ≥30% from baseline for at least 10 s and was accompanied by at least a 3% drop in oxygen levels or an arousal in EEG [24]. The combined number of apneas and hypopneas occurring per hour of sleep constituted the Apnea-Hypopnea Index (AHI). SDB diagnosis was made when the AHI was 5 or greater. We also used AHI as a tool for discrimination between mild (5 ≤ AHI < 15), moderate (15 ≤ AHI < 30), and severe (AHI ≥ 30) sleep apnea [24]. We considered SDB as OSA or CSA if the origin of over half of the recorded respiratory events was obstructive or central, respectively.

LMs were defined as limb movements, each one of which had a duration of 0.5 to 10 s and a minimum amplitude of 8 mV above resting EMG [26,27]. LMs were considered to be part of PLMS only if they occurred during sleep in a sequence of 4 or more movements, which were separated by an interval of more than 5 s and less than 90 s (from limb movement onset to limb movement onset) [28,29]. PLMS index (PLMSI) was defined as the number of PLMs occurring per hour of sleep. In this study, we used a cut-off point of PLMSI ≥15/h for determining patients having clinically significant PLMS [28]. According to PLMSI, PLMS were classified as mild to moderate when 15 ≤ PLMSI < 30 or severe when PLMSI ≥ 30.

In order to evaluate the association of PLMS with SDB, we classified PLMS as SDB-associated, independent, concurrent but not SDB-associated and mixed (independent and SDB-associated). SDB-associated PLMS are constituted mainly by LMs occurring from 0.5 s before the start of a respiratory event to 0.5 s after the respiratory event [29]. However, some LMs occurred earlier than 0.5 s before the start of a respiratory event and lasted throughout the whole respiratory event. The PLMS constituted mainly by such LMs were characterised as concurrent but not SDB-associated. Finally, PLMS were defined as mixed when both independent and SDB-associated LMs were present in a similar proportion.

Sleep stages, apnea/hypopnea events and PLMs were scored manually by a certified expert somnologist (2013 grandparenting examination for Somnologists commissioned by the ESRS) who was blinded to the clinical outcomes. Assessment of all PSGs was carried out according to the American Academy of Sleep Medicine scoring criteria (AASM, scoring manual version 2.6, 2020) [30].

### 2.5. Statistical Analysis

We presented all binary variables as percentages. In cases of normal distributions, we presented continuous variables with their corresponding mean values and standard deviations (SDs), and in cases of skewed distributions, as medians with interquartile ranges (IQRs). We used unpaired *t*-test, Mann–Whitney U-test, χ^2^ test and Fisher exact test for parametric and non-parametric statistical comparisons of categorical and continuous variables, as appropriate.

Associations of baseline characteristics with the presence of PLMS in the acute stroke phase and the likelihood of excellent functional clinical outcome at 3 months (mRS-score: 0–1) among all patients were evaluated using univariable/multivariable binary logistic regression models before and after adjusting for potential confounders. Variables were selected to be included in multivariable analyses based on a *p*-value cutoff of 0.1 or less. We used a backward stepwise selection procedure in order to conduct multivariable analyses. The robustness of the multivariable models was confirmed by repeating all multivariable analyses using a forward selection procedure. We present associations using odds ratios (OR) with corresponding 95% confidence intervals (CI). A *p*-value of 0.05 or less was used in multivariable logistic regression analyses in order to confirm statistical significance. All statistical analyses were performed with the R—software version 3.5.0 (R Foundation for Statistical Computing, Vienna, Austria) [31].

## 3. Results

We screened for eligibility 155 consecutively admitted patients during the four-year study period. Based on the predefined exclusion criteria, 29 patients were excluded from this study (TIA: 7; stroke mimics: 5; current respiratory infection or sepsis: 4; and non-interpretable data from PSG: 13).

Our final cohort comprised 126 consecutively admitted patients {68% men, mean age 60 ± 11 years, median NIHSS-score at initial presentation: 3 (IQR: 2–7)}, within 72 h from symptom onset with AIS (*n* = 109, 87%) or ICH (*n* = 17, 14%). Baseline characteristics of our study participants, including cardiovascular risk factors, neurologic deficit assessment scales, results of the diagnostic work-up during and beyond the hospitalisation, as well as underlying etiologies of the index event according to the TOAST classification, are summarised in Table 1.

In the acute stroke phase, different sleep stages: non-REM stage 1 (N1), 2 (N2) and 3 (N3) constituted approximately 16%, 40% and 15% of the total sleep time, respectively. The remaining 11% was REM sleep stage. The overall rate of PLMS in our cohort was 76%, and the majority of patients with PLMS were men (71%). The rate of PLMS among patients with AIS and ICH was 77% and 71%, respectively. The rates of mild to moderate and severe PLMS among all patients were 16% and 60%, respectively, and the mean PLMSI was 58±39. The rate of SDB among patients with PLMS was 87%. The rates of SDB-associated PLMS, independent PLMS, concurrent but not SDB-associated and mixed (independent and SDB-associated) PLMS were 30%, 50%, 9% and 10% respectively. PLMS detection rates differed significantly (*p*-value: <0.001) according to the severity of sleep-disordered breathing in the acute phase (Table 2). More specifically, the PLMS detection rates were 48%, 69%, and 87% among patients with no, mild, moderate/severe SDB, respectively (*p*-value for linear trend <0.001).

The results from univariable and multivariable analyses regarding the associations of baseline characteristics with the likelihood of PLMS detection are summarised in Table 3. Initial univariable analyses revealed an association between the per 10-year increase in age and the presence of SDB with PLMS detection. However, in multivariable logistic regression models conducted by backward selection analysis, only SDB was independently (*p*-value <0.05) associated with the likelihood of PLMS detection in the acute stroke phase (OR:4.869, 95%CI:1.884–12.784, *p*-value: 0.001). We obtained identical results after repeating the multivariable analyses using the forward selection procedure.

After following all patients for three months, in accordance with the predefined protocol, we demonstrated that baseline stroke severity (OR per 1-point increase in NIHSS-score:0.819, 95%CI:0.737–0.895, *p*-value < 0.001) and PLMS detection (OR:0.099, 95%CI:0.009–0.482, *p*-value = 0.015) were independent predictors for excellent functional outcome (mRS-scores: 0–1) (Table 4).

## 4. Discussion

Our study revealed a high prevalence of PLMS in the acute stroke setting (76%), with 60% of acute stroke patients having severe PLMS (defined as PLMSI ≥ 30/h). SDB was independently associated with higher odds of PLMS. In addition, our analysis showed that besides increasing NIHSS-score on admission, the presence of PLMS is a significant predictor of poor functional outcome at three months.

PLMS prevalence among our acute stroke patients was more than twice as high as the one estimated in a recent meta-analysis, which included ten previous studies (76% versus 32%, respectively) [8,32,33,34,35,36,37,38,39,40]. Moreover, only three of the aforementioned studies evaluated patients during the acute phase of stroke using a PLMSI ≥ 10 or PLMSI ≥ 15 as a cut-off point [33,35,38]. The estimated PLMS prevalence in these studies ranged between 25% and 54.3%. Our higher rates of detected PLMS can be explained by the following facts. First, sleep architecture was remarkably impaired in our patients during the acute phase of stroke, consisting mainly of non-REM sleep stages N1 and N2 [41]. It is well established that PLMS occur most commonly during stages N1 and N2 [42,43], which is also confirmed by the high values of median PLMSI in the N1 and N2 stages found in our analysis (68 and 51, respectively). Moreover, the rate of SDB in our study population was very high (79%), with the majority of patients having severe SDB. A significant association between PLMS and SDB, as well as the increasing AHI, has already been described in the literature [44,45,46,47,48,49]. Additionally, our data analysis demonstrated a clear trend towards detecting PLMS in patients with higher SDB severity (Table 2). As a result, there was a high possibility of detecting SDB-associated PLMS in our stroke patients. Finally, in contrast to our design, all previous studies did not include SDB-associated PLMS during their analysis, which obviously led to lower estimated rates of PLMS.

On the other hand, although SDB was detected in almost 87% of the patients with acute stroke and PLMS, after manually assessing the temporal association between PLMs and respiratory events, we found that the frequency of independent PLMS was much higher than the one of SDB-associated PLMS (50% versus 30%, respectively). This finding is also supported by previous studies showing that the number of PLMS did not sufficiently decrease after CPAP implementation in patients with SDB and PLMS [49,50,51]. Sympathetic overactivity, apart from being present in sleep-disordered patients, has also been linked to the generation of PLMs [52]. Based on these findings, it could be inferred that the presence of SDB leads to a sympathetic overactivity, which in turn generates “genuine” PLMS, creating a vicious circle. Therefore, PLMS and SDB may frequently coexist but are not always directly or causally associated in the acute stroke setting.

As formerly reported in the literature, the vast majority of patients with acute stroke and PLMS in our cohort were men [36,38]. Furthermore, no significant difference was documented regarding the rate of PLMS between AIS and ICH (77% and 71%, respectively). To our knowledge, there is no other study providing evidence about the rate of PLMS among patients with ICH. Risk factors for the presence of PLMS in stroke patients have been so far insufficiently investigated, and the limited data available in the literature seem to be controversial. Based on the results of a prospective study and a meta-analysis, increasing age, hypertension, AHI and BMI were significantly associated with the likelihood of detecting PLMS in patients with acute stroke [9,38]. On the other hand, the same parameters did not emerge as predictors of PLMS according to the results of three other observational studies [33,34,36]. In our study population, none of the baseline characteristics, including the aforementioned factors, except for the presence of SDB, was independently associated with higher odds of PLMS. Differences in study populations, confounders included in the multivariable analyses, the time frame of PSG in the acute stroke stage and ethnic differences may account for these discrepant results.

Increasing age did not reach statistical significance as a risk factor for PLMS in our analysis, although there was a trend towards detecting PLMS in older patients. In contrast to previous studies supporting a possible association between PLMS pathophysiology and brainstem dysfunction [53,54,55], our results did not demonstrate brainstem localisation of stroke as an independent predictor of PLMS. Moreover, although PLMS are considered responsible for causing frequent arousals and thus sleep fragmentation and daytime sleepiness [2,56,57], in our stroke population, we confirmed that abnormal ESS score was not significantly associated with the possibility of detecting PLMS [37]. Finally, as previously observed, no significant relationship between ischemic stroke aetiology and PLMS was detected in the present study [8].

With regard to functional outcome at three months in patients with acute stroke, our analysis demonstrated that both increasing stroke severity and the presence of clinically significant PLMS (assessed by NIHSS and PLMSI, respectively) are independent predictors of poor functional outcome (assessed by mRS). The negative effect of PLMS on post-stroke recovery is also supported by a prospective study of 24 consecutive patients, which used the Barthel scale for outcome evaluation [58]. To the best of our knowledge, this is the first study reporting a negative impact of on stroke outcome, assessed by mRS. Given the heterogeneity of the ESUS type of ischemic stroke regarding the underlying aetiology, we focused on investigating its potential effect on post-stroke recovery. In contrast to the results of some previous studies reporting favourable outcomes in the case of ESUS type [59,60], we found no significant association of the ESUS type with the likelihood of excellent functional outcome (mRS-score 0–1) at three months among all patients.

Important strengths of our study are the use of a full PSG setting on patients at the hospital wards during the hyperacute stroke phase and the manual assessment of PSG studies, which allowed us to investigate individually the temporal association between PLMS and respiratory events and determine the origin of PLMS. However, there are also certain limitations that should be acknowledged for an accurate interpretation of our results. First, the size of our sample was small, and the NIHSS-score on admission was rather low compared to one of unselected AIS patients admitted in our tertiary stroke centre during the study period (median NIHSS-score 3 versus 7, respectively). Moreover, there was a predominance of male patients and a limited number of patients with ICH included. In addition, patients with severe stroke, disordered comprehension, or altered mental status were excluded. Therefore, our results may not be reproducible in all stroke patients. Furthermore, our sleep evaluation did not entail the recording of RERAs as respiratory events that probably would have increased the rate of SDB-associated type of PLMS. Finally, the routine blood tests performed on our patients did not include serum ferritin levels and thus, iron deficiency was not ruled out as a possible cause of PLMS.

PSG devices are not widely available in stroke centres. Moreover, current guidelines for secondary stroke prevention do not recommend the implementation of PSG in the routine work-up after a stroke. However, if our results are confirmed by larger clinical trials, we believe that PSG should be included in everyday clinical practice of stroke work-up in order to achieve early SDB and PLMS detection and improve post-stroke recovery.

In conclusion, PLMS prevalence and severity in acute stroke patients is very high. SDB is the only significant risk factor for PLMS presence in the acute stroke phase. However, “genuine” PLMS is the predominant type when evaluating association with SDB. In addition, increasing stroke severity and clinically significant PLMS can independently predict a poor post-stroke recovery within three months after the index event. The common presence of mostly severe PLMS in patients with acute stroke and their negative effect on stroke outcomes point out the necessity for early PLMS detection and treatment.

## Figures and Tables

**Table 1:** Clinical characteristics and sleep disorders of our study population (N = 126).

Variable	Overall
Age (years), mean (SD )	60.3 (10.9)
Sex—Male, *n* (%)	85 (67.5%)
BMI, mean (SD )	29.3 (5.7)
Obese, *n* (%)	47 (37.3%)
Intracerebral Hemorrhage, *n* (%)	17 (13.5%)
Ischemic Stroke, *n* (%)	109 (86.5%)
Acute reperfusion therapies, *n* (%)	16 (12.7%)
Neurologic Deficit	
NIHSS_adm_–Score, median (IQR)	3 (2–7)
NIHSS_dis_–Score, median (IQR)	2 (0–2)
mRS at Discharge	
excellent–poor, *n* (%)	75 (59.5%)–51 (40.5%)
mRS at 3 months excellent–poor, *n* (%)	86 (68.3)–40 (31.7)
Comorbidities	
Hyperlipidemia, *n* (%)	48 (38.1%)
Arterial Hypertension, *n* (%)	77 (61.1%)
Diabetes Mellitus, *n* (%)	32 (25.4%)
Congestive Heart Failure, *n* (%)	12 (9.5%)
History of Stroke/Transient Ischemic Attack, *n* (%)	22 (17.5%)
History of Myocardial Infarction, *n* (%)	25 (19.8%)
PFO, *n* (%)	7 (5.6%)
Atrial Fibrillation, *n* (%)	20 (15.9%)
Reduced Ejection Fraction ± Dilated Left Atrium, *n* (%)	3 (2.4%)
Intracardiac Thrombus, *n* (%)	1 (<1%)
Mechanical Valve, *n* (%)	1 (<1%)
Current Smoking, *n* (%)	64 (50.8%)
Excessive Alcohol Intake, *n* (%)	13 (10.3%)
Ischemic stroke Classification	
Non-Cryptogenic, *n* (%)	75 (59.5%)
Cryptogenic ESUS, *n* (%)	33 (26.2%)
Cryptogenic non-ESUS, *n* (%)	18 (14.3%)
Sleep stages	
Ν1, % (Τotal Sleep Time), mean (SD )	16.3 (11.0)
Ν2, % (Τotal Sleep Time), mean (SD )	39.9 (13.5)
Ν3, % (Τotal Sleep Time), mean (SD )	14.5 (9.8)
REM, % (Τotal Sleep Time), mean (SD )	10.8 (6.6)
Sleep-disordered Breathing—SDB, *n* (%)	99 (78.6)
Epworth—Sleepiness Scale score	
0–9 points, *n* (%)	57 (59.4%)
≥10 points, *n* (%)	39 (40.6%)
Periodic Limb Movements during Sleep—PLMS, *n* (%)	96 (76.2%)
Periodic Limb Movements during Sleep (PLMS) N = 96	
Sex—Male, *n* (%)	68 (70.8%)
Stroke type	
Intracerebral Hemorrhage, *n* (%)	12 (70.6%)
Ischemic Stroke, *n* (%)	84 (77%)
PLMS Severity	
Mild to Moderate, *n* (%)	20 (15.9%)
Severe, *n* (%)	76 (60.3%)
PLMSI, mean (SD )	58.0 (39.3)
PLMSI in Sleep stages	
PLMSI_N1, median (IQR)	67.5 (37.2–103.1)
PLMSI_N2, median (IQR)	51.3 (33.1–74.1)
PLMSI_N3, median (IQR)	20.5 (1.8–60.8)
PLMSI_REM, median (IQR)	9.8 (0.0–34.9)
Sleep-disordered Breathing (SDB), *n*(%)	83 (86.5)
Classification of SDB	
Mild, *n* (%)	11 (13.3%)
Moderate, *n* (%)	21 (25.3%)
Severe, *n* (%)	51 (61.4%)
Classification of PLMS	
SDB-associated, *n* (%)	29 (30.2%)
Independent of SDB, *n* (%)	48 (50.0%)
Concurrent but not SDB-associated, *n* (%)	9 (9.4%)
Mixed (Independent and SDB-associated), *n* (%)	10 (10.4%)
Type of SDB	
Obstructive, *n* (%)	66 (79.5%)
Central, *n* (%)	17 (20.5%)
Epworth—Sleepiness Scale score	
0–9 points, *n* (%)	42 (59.2%)
≥10 points, *n* (%)	29 (40.8%)
Apnea-Hypopnea Index (AHI), mean (SD)	36.8 (24.6)

SD—Standard Deviation, IQR—Interquartile Range, BMI—Body Mass Index, NIHSSadm—NIHSS Score on admission, NIHSSdis—NIHSS Score at discharge, mRS—modified Rankin Scale, PFO—Patent Foramen Ovale, ESUS—Embolic Stroke of Undetermined Source, REM—Rapid Eye Movement, SDB—Sleep-disordered Breathing, PLMS—Periodic Limb Movements during Sleep, PLMSI—PLMS index.

**Table 2:** Prevalence of PLMS detection in the acute stroke stratified by the severity of Sleep-Disordered Breathing severity.

Sleep Disordered Breathing Severity	PLMS Detection (−)	PLMS Detection (+)	*p*-Value	*p*-Value for Linear Trend
No	52%	48%	<0.001	<0.001
Mild	31%	69%		
Moderate or Severe	13%	87%		

**Table 3:** Univariable and multivariable logistic regression analyses depicting the associations of baseline characteristics with the likelihood of detecting PLMS among all patients.

Variable	Univariable Logistic Regression Analysis		Multivariable Logistic Regression Analysis	
	Odds Ratio (95% CI)	*p*-Value *	Odds Ratio (95% CI)	*p*-Value *
Age (per 10-year increase)	1.572 (1.066–2.376)	0.026	1.373 (0.922–2.089)	0.126
Male (Sex)	1.857 (0.789–4.328)	0.151		
Obese (ΒΜΙ > 30 kg/m^2^)	1.882 (0.784–4.896)	0.171		
ICH vs. AIS	0.714 (0.239–2.419)	0.561		
NIHSS-Score (per 1-point increase)	1.079 (0.988–1.206)	0.127		
Hyperlipidemia	1.083 (0.469–2.593)	0.854		
Arterial Hypertension	1.275 (0.548–2.924)	0.568		
Diabetes Mellitus	1.486 (0.572–4.373)	0.438		
Congestive Heart Failure	0.931 (0.256–4.417)	0.919		
History of Stroke/TIA	0.469 (0.177–1.304)	0.134		
History of Myocardial Infarction	2.676 (0.839–11.939)	0.133		
PFO	0.391 (0.081–2.086)	0.238		
Atrial Fibrillation	3.231 (0.857–21.142)	0.131		
Brainstem Stroke Location	2.280 (0.705–10.246)	0.212		
Current Smoking	1.200 (0.612–2.421)	0.601		
Excessive Alcohol Intake	1.047 (0.295–4.915)	0.948		
ESUS	0.815 (0.476–1.429)	0.463		
Sleep-disordered breathing (SDB)	5.587 (2.229–14.364)	<0.001	4.869 (1.884–12.784)	0.001
Central (vs. Obstructive) SDB	1.116 (0.315–5.262)	0.875		
Epworth-Sleepiness-Scale Score > 9	1.036 (0.412–2.683)	0.941		

* A cutoff of *p* < 0.1 was used for the selection of candidate variables for inclusion in multivariable logistic regression models. BMI—Body Mass Index, ICH—Intracerebral Hemorrhage, AIS—Acute Ischemic Stroke, NIHSS—National Institutes of Health Stroke Scale, TIA—Transient Ischemic Attack, PFO—Patent Foramen Ovale, ESUS: Embolic Stroke of Undetermined Source.

**Table 4:** Univariable and multivariable logistic regression analyses depicting the associations of baseline characteristics with the likelihood of excellent functional outcome (mRS-score 0–1) at 3 months among all patients.

Variable	Univariable Logistic Regression Analysis		Multivariable Logistic Regression Analysis	
	Odds Ratio (95% CI)	*p*-Value *	Odds Ratio (95% CI)	*p*-Value *
Age (per 10-year increase)	0.840 (0.588–1.188)	0.328		
Male (Sex)	0.842 (0.366–1.870)	0.678		
Obese (ΒΜΙ > 30 kg/m^2^)	0.725 (0.336–1.573)	0.411		
ICH vs. AIS	1.603 (0.524–6.004)	0.437		
NIHSS (per 1-point increase)	0.825 (0.747–0.898)	<0.001	0.819 (0.737–0.895)	<0.001
Hyperlipidemia	1.038 (0.482–2.280)	0.925		
Arterial Hypertension	0.568 (0.249–1.243)	0.165		
Diabetes Mellitus	1.031 (0.442–2.525)	0.944		
Congestive Heart Failure	0.923 (0.272–3.641)	0.901		
History of Stroke/TIA	2.382 (0.815–8.717)	0.141		
History of Myocardial Infarction	0.416 (0.168–1.027)	0.055	0.883 (0.297–2.730)	0.825
PFO	0.602 (0.127–3.181)	0.520		
Atrial Fibrillation	1.102 (0.404–3.337)	0.855		
Reduced EF ± Dilated LA	0.929 (0.086–20.333)	0.952		
Brainstem–Stroke	0.500 (0.195–1.302)	0.149		
Current Smoking	1.108 (0.598–2.083)	0.746		
Excessive Alcohol Intake	0.718 (0.223–2.519)	0.584		
ESUS	1.682 (0.974–3.090)	0.075	1.647 (0.880–3.258)	0.131
Sleep-disordered breathing (SDB)	0.416 (0.130–1.118)	0.103		
Central (vs. Obstructive) SDB	0.7788 0.2864 2.2013	0.627		
Apnea-Hypopnea Index	0.982 (0.967–0.997)	0.022	0.984 (0.966–1.002)	0.094
Epworth-Sleepiness-Scale Score > 9	0.828 (0.329–2.114)	0.689		
PLMS	0.109 (0.017–0.392)	0.004	0.099 (0.009–0.482)	0.015

* A cutoff of *p* < 0.1 was used for the selection of candidate variables for inclusion in multivariable logistic regression models. BMI: Body Mass Index, ICH: Intracerebral Hemorrhage, AIS: Acute Ischemic Stroke, NIHSS: National Institutes of Health Stroke Scale, TIA: Transient Ischemic Attack, PFO: Patent Foramen Ovale, ESUS: Embolic Stroke of Undetermined Source, PLMS: Periodic Limb Movements during Sleep.

## Data Availability

The data that support the findings of this study are available from the corresponding author (A.B.) upon reasonable request.
